# Metformin in Gestational Diabetes Mellitus: To Use or Not to Use, That Is the Question

**DOI:** 10.3390/ph16091318

**Published:** 2023-09-18

**Authors:** Vera Tocci, Maria Mirabelli, Alessandro Salatino, Luciana Sicilia, Stefania Giuliano, Francesco S. Brunetti, Eusebio Chiefari, Giovambattista De Sarro, Daniela P. Foti, Antonio Brunetti

**Affiliations:** 1 Department of Health Sciences, University “Magna Græcia” of Catanzaro, 88100 Catanzaro, Italy; tocci.vera@gmail.com (V.T.); maria.mirabelli@unicz.it (M.M.);; 2 Operative Unit of Endocrinology, Diabetes in Pregnancy Ambulatory Care Center, Renato Dulbecco University Hospital, 88100 Catanzaro, Italy; 3 Department of Experimental and Clinical Medicine, University “Magna Græcia” of Catanzaro, 88100 Catanzaro, Italy; foti@unicz.it

**Keywords:** gestational diabetes mellitus, metformin, guidelines, fetal development, transgenerational effects

## Abstract

In recent years, there has been a dramatic increase in the number of pregnancies complicated by gestational diabetes mellitus (GDM). GDM occurs when maternal insulin resistance develops and/or progresses during gestation, and it is not compensated by a rise in maternal insulin secretion. If not properly managed, this condition can cause serious short-term and long-term problems for both mother and child. Lifestyle changes are the first line of treatment for GDM, but if ineffective, insulin injections are the recommended pharmacological treatment choice. Some guidance authorities and scientific societies have proposed the use of metformin as an alternative pharmacological option for treating GDM, but there is not yet a unanimous consensus on this. Although the use of metformin appears to be safe for the mother, concerns remain about its long-term metabolic effects on the child that is exposed in utero to the drug, given that metformin, contrary to insulin, crosses the placenta. This review article describes the existing lines of evidence about the use of metformin in pregnancies complicated by GDM, in order to clarify its potential benefits and limits, and to help clinicians make decisions about who could benefit most from this drug treatment.

## 1. Introduction

Gestational diabetes mellitus (GDM), defined as any degree of glucose intolerance that develops or is first recognized during pregnancy, is a common gestational endocrine disorder that can cause short- and long-term health problems for both mothers and their fetuses [1]. In 2018, the World Health Organization has classified GDM as a “global health research priority” due to its increasing prevalence and its association with many adverse maternal-fetal occurrences, such as pre-eclampsia and eclampsia, pre-term delivery, caesarean section, macrosomia, neonatal hypoglycemia, neonatal respiratory distress syndrome, as well as an increased risk of developing type 2 diabetes (T2D), obesity, and cardiovascular disease after gestation [1]. A robust meta-analysis conducted by Ye and colleagues has recently quantified the risk of short-term adverse pregnancy outcomes associated with GDM across a sample size of 7,506,061 pregnancies [2]. The key findings from this analysis are presented in Table 1.

It is estimated that one in every six pregnant women develops GDM globally [3]. However, the prevalence of GDM varies greatly among different ethnic groups and different screening methods and glucose thresholds used for diagnosing this condition [4]. Hispanic, African American, Native American, South Asian, and Pacific Islander women have the highest prevalence rates of GDM [1,2,3,4], together with women living in the Southern Mediterranean region, where the rate of GDM diagnosis reaches 28% [5,6]. Obesity is the major risk factor for GDM, as nearly 50% of obese women develop this condition [7]. In this regard, in recent years there has been a trend for women of reproductive age to gain more body weight, and this is believed to represent one of the key clinical factors behind the observed increased prevalence rates of GDM across continents, together with the current demographic trend of a delayed motherhood [6]. It is the recent switch from a stringent two-step screening process (using Carpenter-Coustan glucose thresholds, originally calculated based on maternal risk of developing T2D after gestation) to a universal single-step approach the critical factor that has nearly doubled the incidence rate of GDM in several European and Australasian countries [8]. The new, single-step approach, promoted by the International Association of Diabetes and Pregnancy Study Groups (IADPSG), uses lower glucose thresholds that are calculated based on the risk of adverse pregnancy outcomes instead of maternal T2D in the postpartum period [9,10].

In fact, the primary concern raised by critics of the IADPSG diagnostic criteria is the notable increase in cases of GDM among pregnant women. In the United States, a group of experts convened by the National Institutes of Health in 2013 projected that adopting the IADPSG approach would result in a prevalence of GDM ranging from 15 to 20%, compared with 5–6% with the Carpenter-Coustan criteria [11]. Nonetheless, recent international data on GDM prevalence indicate significant disparities, with rates ranging from 6.6% in Japan and Nepal to 45.3% of pregnancies in the United Arab Emirates, underscoring ethnic variations in the risk of GDM [12]. Regardless of how the condition is diagnosed, the recommended therapeutic options for the standard care of GDM are lifestyle changes (i.e., medical nutrition therapy associated with physical activity) and insulin injections. These interventions can significantly reduce the risk of adverse pregnancy outcomes for the affected mothers and their infants [4]. Although metformin has the potential to counteract gestational insulin resistance (which stands at the core of GDM pathophysiology), there is much uncertainty about its use during pregnancy, so that international guidelines differ in their recommendations. In order to assess the potential benefits and drawbacks of using metformin during pregnancy, it is necessary to have a clear understanding on how GDM develops and leads to adverse maternal-fetal outcomes, as well as on the different mechanisms underpinning metformin action. This review article discusses the potential benefits of metformin for pregnant women, the potential risks associated with its use, and the questions that remain unanswered by current research.

## 2. Pathophysiology of Gestational Insulin Resistance and Gestational Diabetes Mellitus (GDM)

GDM typically develops when there is β-cell dysfunction in a pregnant woman who is (para)physiologically resistant to insulin. From the beginning of pregnancy, the maternal organism undergoes many physiological changes to support the correct development of the fetus. These changes primarily involve the metabolism of nutrients and the consequent adaptations of maternal insulin sensitivity, which can shift throughout the course of gestation to properly meet fetal demands. A growing fetus has a high energy requirement and uses glucose at an average rate of 6 mg/kg/min, which is nearly three times the adult requirement of 2 mg/kg/min [13]. In normal pregnancies, most of this high fetal glucose requirement is maintained by a continuous transplacental transfer from the mother to the fetus. In fact, the blood fetal glucose concentrations are directly proportional to maternal glycemic levels [13]. During early pregnancy (up to 20 weeks of gestation), the mother’s organism becomes more sensitive to insulin in order to build up energy stores for the fetus (anabolic phase of pregnancy) [14,15]. This is due to the action of estrogens, which cause the maternal pancreas to undergo β-cell hyperplasia and produce more insulin [16]. As a result, the mother’s organism stores more glycogen in the liver and muscle in preparation for the later stages of pregnancy. Then, as the pregnancy progresses, many counter-regulatory hormones (such as cortisol, leptin, progesterone, placental lactogen, and growth hormones) reverse maternal insulin sensitivity by inducing insulin resistance (catabolic phase of pregnancy). This state of insulin resistance is functional in order to reduce maternal glucose utilization peripherally and to increase glucose transport across the placenta, providing the fetus with readily available energy [13,14,15]. At this point, glucose homeostasis is ensured by maternal β-cell hypertrophy and hyperplasia, mainly supported by prolactin stimulation [17].

**Figure 1:**
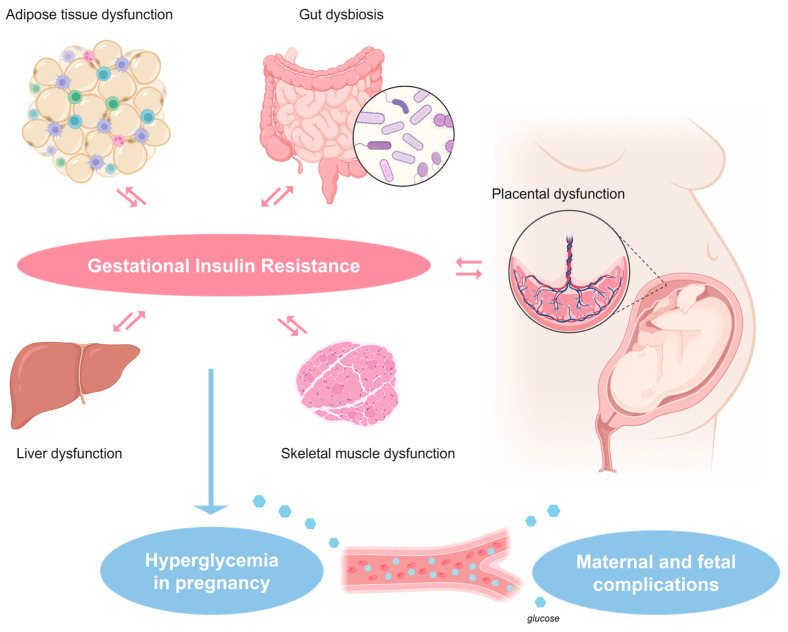
Maternal and placental factors implicated in the pathophysiology of gestational insulin resistance, which can potentially be addressed with the use of metformin in women diagnosed with gestational diabetes mellitus.

If these metabolic and hormonal adaptations occur correctly during pregnancy, women are able to maintain normoglycemia; vice versa if these adaptations do not occur, because of single or a combination of clinical/environmental factors affecting insulin secretion and/or sensitivity (Figure 1), GDM rises and develops.

GDM has been described to be linked with a decrease in hepatic suppression of glucose production [18]. This phenomenon is not only due to inadequate glucose sensing, but also to the increased hepatic delivery of substrates that can be used for gluconeogenesis (i.e., proteins derived from dietary intake or breakdown of muscle stores), a metabolic pathway not entirely dependent on insulin activity [18]. Therefore, the liver does not seem to be the main driver of glucose intolerance in women with GDM [19]. The main site affected by gestational insulin resistance is skeletal muscle, which considerably reduces its sensitivity to insulin in the second half of pregnancy [20]. Skeletal muscle insulin-resistance can explain the 50–60% reduction in whole-body insulin-stimulated glucose-uptake that is observed in pregnant women during late gestation [18,19,20]. The level of expression of insulin receptor (IR) on skeletal muscle is the same before and after pregnancy, suggesting that gestational insulin resistance is mediated by altered post-receptor signal transduction mechanisms [20]. The main IR second messenger involved in these molecular alterations is the insulin receptor substrate-1 (IRS1), that, following tyrosine phosphorylation, binds to the P85 subunit of phosphatidylinositol 3 kinase (PI3K) and activates the downstream insulin pathway signaling that promote glucose uptake in myocytes and adipocytes via the glucose transporter GLUT4 [21,22]. IRS-1 expression is almost halved in the skeletal muscle of obese pregnant women and women with GDM with respect to nonpregnant obese women, suggesting a key role for this molecular factor in generating gestational insulin resistance [23]. Although the precise molecular mechanisms underlying gestational insulin resistance are unknown, it is hypothesized that proinflammatory factors and adipokines which negatively impact on IR signaling may be involved, as for the more common form of insulin resistance that is associated with obesity [20,21,24]. One investigated mechanism involves the aberrant phosphorylation of IRS-1 on threonine/serine residues induced by cytokines and cytokine signaling. While the tyrosine phosphorylation of IR and IRS-1 causes activation of IR, phosphorylation of threonine/serine residues results in signal inhibition [20,21]. The aberrant signaling of IR is thought to play a key role in the development of gestational insulin resistance, as well as in T2D, obesity, and metabolic syndrome [25]. Furthermore, women with a history of GDM often have evidence of low-grade inflammation, suggesting that the sustained activation of intracellular proinflammatory pathways by circulating cytokines, such as TNFα and interleukins (ILs) released by a dysfunctional placenta and/or an enlarged visceral fat may be the cause of the exacerbated insulin resistance seen during pregnancy [26]. Our group has recently presented clinical evidence indicating that an elevated pre-pregnancy body mass index (BMI) is the most influential modifiable factor in the development of GDM compared with other risk factors that cannot be modified (i.e., advanced maternal age). This conclusion is based on a range of potential mechanisms that may contribute to this relationship [6]. The NLRP3 inflammasome is a crucial component of the innate immune response and plays a pivotal role in regulating the extracellular release of cytokines. It consists of NLRP3 as the sensor, ASC as the adaptor, and caspase-1 as the effector [27]. Various microbial motifs and danger signals can activate the NLRP3 inflammasome within immunocompetent cells. Upon activation, the NLRP3 inflammasome assembles and stimulates the production and release of proinflammatory cytokines, specifically IL-1β and IL-18, by activating caspase-1 [28]. Recent research suggests that hyperactivation of the NLRP3 inflammasome contributes to the development of systemic inflammatory disorders like T2D and GDM. Metabolic stressors, such as an excessive intake of free fatty acids from the diet or their release from an enlarged adipose tissue, can trigger the activation of the NLRP3 inflammasome [29]. Additionally, some studies have found that pregnancy is physiologically associated with a lower post-meal response in terms of glucagon-like peptide 1 (GLP-1) secretion. This reduced response tends to return to normal after delivery. However, in pregnant women with GDM, the loss of the incretin effect is more pronounced and tends to persist after giving birth, and this may be a risk factor for developing obesity, T2D, and metabolic syndrome in the post-partum period [30].

## 3. Metformin

For most patients with T2D, metformin is the recommended first-line pharmacological treatment [31]. Metformin represents one of the oldest and most widely used oral antihyperglycemic agents worldwide, either alone or in combination with other antidiabetic medications, including insulin [31,32,33]. Metformin is an insulin-sensitizer belonging to the biguanides class of drugs, which comprises molecules with two linked guanidine rings in their chemical structures. Phenformin and buformin are biguanides with antidiabetic effects that are similar to metformin, but they have been withdrawn by the American Food and Drug Administration (FDA) in the 1970s because of the high risk of lactic acidosis and cardiovascular diseases associated with their use [34]. The occurrence of lactic acidosis with metformin is a rare event (estimated 6.3 cases per 100,000 person-years) [35], but because of this remote possibility, metformin is contraindicated in conditions harboring a high risk of acidosis, such as severe respiratory failure, heart failure (NYHA class III–IV), or severe liver insufficiency. Metformin is also contraindicated in patients who are at risk of acute renal failure (i.e., because of surgery, use of iodized contrast media, and dehydration) or have chronic kidney disease with an estimated glomerular filtration rate less than 30 mL/min [36]. Metformin is generally well-tolerated by patients. It has a modest risk of gastrointestinal side effects, but the modern formulations that are released slowly over 24 h are generally compatible with a better gastrointestinal tolerance than the traditional formulations (total gastrointestinal adverse events: 9.2% vs. 19.8%, diarrhea: 3.1% vs. 13.5% [37]). Metformin is not associated with hypoglycemia or weight gain, which are typical side effects of conventional antidiabetic medications (i.e., insulin secretagogues/sulfonylureas, insulin) [31,38]. As concerns the clinical benefits of using metformin, in the landmark United Kingdom Prospective Diabetic Study (UKPDS), this drug has been shown to reduce the risk of myocardial infarction associated with T2D by 39%, and the risk of coronary deaths by 50% [39], whereas observational studies have evidenced a reduction in cancer risk in T2D patients that take metformin as their antidiabetic medication [22].

Despite being in use for over 60 years, the molecular mechanisms by which metformin effectively reduce hyperglycemia in T2D and/or mitigate cancer risk are not yet fully understood. In recent years, many authoritative research groups have placed mitochondria at the heart of metformin’s cellular actions [40]. In vitro studies on intact cells and isolated mitochondria have suggested that metformin accumulates in mitochondria causing the inhibition of the respiratory-chain complex 1 [41,42]. The inhibition of the respiratory-chain complex 1 reduces the ability of cells to produce ATP, causing the indirect activation of AMPK, an energy sensor that is triggered by cellular energy depletion. In turn, activation of AMPK switches cellular metabolism to a catabolic state, resulting in improved fatty acid metabolism via the inhibitory phosphorylation of the acetyl CoA carboxylase, and reduces the synthesis of gluconeogenetic enzymes [43]. These effects are particularly relevant in the liver, as most of the anti-hyperglycemic effects of this drug are thought to be due to reduced glucose output and improved hepatic insulin sensitivity [44]. On the other hand, hepatic activation of AMPK is not sufficient to explain all the clinical effects linked to metformin, which include, but are not limited to improved liver functions. Some studies suggest that this drug may interact with the gut-incretin axis to promote an increase in GLP-1 secretion [45]. Additionally, metformin is supposed to exert a wide range of anti-inflammatory and anti-neoplastic effects because of the downregulation of expression of multiple cytokines. These effects seem to be independent from AMPK activation and may be due to the ability of metformin to prevent the translocation of NF-κB to the nucleus, given that this drug has been shown to inhibit the phosphorylation of IκB and IKKα/β in different cell types [46]. Furthermore, metformin is thought to possess antioxidant and antithrombotic activities, as it reduces the production of reactive oxygen species (ROS) caused by mitochondrial dysfunction in vascular endothelial cells, while it improves, at the same time, the generation of nitric oxide (NO) by the endothelial NO synthase (eNOS) [47,48]. Finally, and even more pertinent to the core issue of this review, there is a large body of evidence indicating that metformin is an effective treatment for polycystic ovary syndrome (PCOS) and related female infertility. Many studies have shown that metformin can improve ovarian insulin sensitivity and reduce clinical and biochemical hyperandrogenic traits in infertile women affected by PCOS, while increasing their ovulation and conception rates [49,50]. Consequently, the number of women being prescribed metformin to treat PCOS has become high, and thus, the onset and progression of pregnancy in women under metformin treatment are now common findings in the obstetrical and endocrinological clinical settings.

Very recently, some research groups, including ours, have highlighted a direct modulatory role of metformin in nuclear events that regulate the function of transcription factors and chromatin-remodeling proteins [51,52]. These include the hypoxia-inducible factor 1α (HIF-1α), which is known to modulate the transcription of hypoxia-related genes relevant to inflammation [53], and the high-mobility group A1 (HMGA1) chromatin-remodeling protein [54], which is involved in a variety of metabolic, embryological, and oncological processes [55,56,57]. Exposure to metformin (either at pharmacological or supra-pharmacological concentrations) has been shown to downregulate the nuclear HMGA1 protein in male germ tumor and hepatoma cell lines, leading to reduced cell viability, cell migration and proliferation [54]. Whether these effects of metformin can be observed in non-tumor cells is yet unknown. However, fetal progenitor cells and cancer cells share some similarities, including the hyperactivation of HIF-1α and HMGA1-related biological pathways [58,59]. This has led us to speculate that in utero exposure to metformin, causing molecular interferences with these nuclear factors, might be detrimental for normal fetal development [54].

Currently, there is limited information available on how metformin affects fetal tissues and cells, as well as the functions and metabolism of placental trophoblasts. The effects of administering metformin at adult-level doses to fetal livers may differ due to the underdeveloped metabolic state and reduced antioxidant defenses of hepatocytes in the fetal stage. Although fetal hepatocytes do not engage in gluconeogenesis until birth because glucose is received from the mother, inhibition of the flux of substrates involved in gluconeogenesis by metformin may impact several anabolic pathways. In fetal hepatocytes, metformin activates AMPK, inhibits mTOR, decreases glucose production, and reduces oxygen consumption, similar to its effects in children’s and adults’ hepatocytes [60]. However, specifically in fetal hepatocytes, the administration of metformin causes negative effects on the activation of stress pathways and the synthesis of growth factors (such as IGFs and related binding proteins, which are, interestingly, transcriptionally regulated by HMGA1 [61]). These findings, recently obtained from experiments conducted on primary hepatocytes derived from fetal and juvenile macaques, emphasize the need for further research on the potential harm to offspring’s metabolism caused by exposure to metformin during pregnancy [60].

Although the exact underlying mechanisms are not yet fully understood, disruptions in NLRP3 inflammasome-related processes have been suggested to play a role in the development of placental inflammation and pre-eclampsia, both of which are observed more frequently in women with GDM than in women with normal glucose tolerance [62]. Placental trophoblasts, which are epithelial cells with immune competence, constitute most of the maternal-fetal interface and are vital for the achievement of full-term pregnancy. Research has shown that there is an increased expression of NLRP3 and associated molecules (such as caspase 1 and IL-1β), indicating excessive activation of the NLRP3 inflammasome, in placentas from women with pre-eclampsia [63]. Additionally, dysfunctional mitochondria have been observed in their placental tissues [64]. The shift in glucose metabolism to glycolysis in pre-eclamptic placental trophoblasts has been linked to activation of the NLRP3 inflammasome and cell death (i.e., NLRP3 inflammasome-induced pyroptosis). In recent in vitro studies conducted on immortalized trophoblasts (specifically, the HTR-8/SVneo cell line), metformin has effectively suppressed excessive glycolysis and enhanced mitochondrial function, also mitigating the activation of the NLPR3 inflammasome within these cells [65]. In contrast, an analysis of primary human trophoblasts obtained from term placentas revealed a reduction in oxidative phosphorylation following treatment with metformin. This decline was attributed to the inhibition of respiratory-chain complex 1, although there may be other underlying mechanisms involved. Furthermore, the introduction of metformin to these trophoblasts led to a dose-dependent accumulation of lipids, specifically long-chain polyunsaturated fatty acids, due to the drug’s negative impact on lipid uptake and/or oxidation. However, further research is required to explore the effects of metformin on the availability and delivery of fatty acids to the fetus, which are crucial for proper growth and development [66].

## 4. Treatment Options for GDM

According to international guidelines, mothers who have been diagnosed with GDM should immediately start a personalized medical nutritional plan and physical activity in order to maintain fasting and post-prandial maternal blood glucose levels within the recommended targets [1,4]. The primary goal of managing GDM is to achieve glucose levels that are close to normal, as this measure can help prevent excessive fetal growth/adiposity [67]. One of the main approaches to control glucose levels is by adjusting the quality and distribution of carbohydrates in the diet (i.e., preferring low-to-medium glycemic index carbohydrates and increasing the number of meals). Although there is currently limited data on a specific dietary protocol for GDM [4], international guidelines recommend a carbohydrate intake ranging from 33% to 55% of total energy intake. Studies have shown improved pregnancy outcomes with both lower and higher carbohydrate intake, as long as regular nutrition counseling and clinical follow-ups with qualified healthcare professionals are provided [68]. Generally, the nutrition requirements for women with GDM are similar to those without GDM, and it is generally agreed upon that carbohydrate intake should not be limited to less than 175 g per day. However, special attention is needed for dietary modifications in women with GDM to ensure healthy and mindful eating, maintain normal glucose levels, prevent drastic fluctuations in glucose levels, and achieve appropriate gestational weight gain, which depends on the pre-pregnancy BMI according to the Institute of Medicine (IOM) recommendations [69]. Once GDM is diagnosed, implementing exercise interventions of mild to moderate intensity, including aerobic or resistance training, at a frequency of at least three sessions per week, can also be beneficial for managing glycemic control and enhancing maternal and fetal outcomes. Engaging in exercise and incorporating more physical activity into daily living enhance maternal insulin sensitivity and promote muscular glucose uptake [70]. In most cases (85%), these lifestyle changes are sufficient to achieve glycemic goals during pregnancy [1,4]. In the remaining cases, where a pharmacological treatment approach is needed, insulin, in form of an injective regimen, is usually considered as the first choice. However, there can be barriers to insulin therapy during pregnancy, such as the cost, availability, fear of needles and/or hypoglycemia, and patient preference. In these cases, an oral medication can represent an alternative solution [4]. Although individual randomized control clinical trials (RCTs) support the efficacy of metformin or the insulin secretagogue glyburide in reducing glucose levels in women with GDM, these drugs are not recommended as first-line pharmacological therapies by many scientific societies and guidance authorities. The main reason is that these antidiabetic medications cross the placenta and may impair fetal growth and development, regardless of whether maternal glycemic control is achieved or not [4].

In the following sections, we evaluated the benefits and drawbacks of metformin use during pregnancy, as well as the heterogeneous positions of scientific societies and guidance authorities on GDM pharmacological treatment. The effects of metformin in pregnancy on both mother and fetus have been considered, taking into account any potential short- and long-term consequences.

## 5. Benefits of Metformin Use in Pregnancies Complicated by GDM

Metformin, which increases cell sensitivity to insulin, is without doubt, a reasonable pharmacological strategy for treating diseases whose pathophysiology is characterized by insulin resistance. Metformin is a cheaper option than insulin and can be taken orally, which may be more appealing to patients than injecting insulin [71]. At physiological pH, metformin exists in the form of cationic species (>99.9%) [72]. The intestinal absorption, tissue distribution, and renal elimination of metformin are mainly mediated by organic cation transporters (OCTs), which are Na+-independent electrogenic channels located in plasma membranes. OCTs are broadly expressed in fetal and in placental tissues, resulting in fetal metformin concentrations that are equal to maternal levels [73,74]. The expression and functional plasma membrane localization of OCTs is limited in embryos, which can result in minimal exposure to metformin at this developmental stage [75]. Starting from the second trimester of pregnancy, there is a fine-tuned increase in OCT expression, which becomes critical for assuring an adequate transport of nutrients to the growing fetus, and this phenomenon may lead to a higher exposure to metformin [75,76].

There is no clear evidence to date that metformin is teratogenic during pregnancy, and studies investigating its use have been reassuring [77]. Therefore, the use of metformin during the first trimester of pregnancy is generally considered safe. In the EUROmediCAT population-based registry study, there was no increased risk of non-genetic congenital abnormalities associated with exposure to metformin, after adjusting for maternal age, registry, multiple births, and maternal diabetes status (pre-existing diabetes or GDM) [78]. The only significant positive association was found for pulmonary valve atresia, which is likely due to chance because of the multiple testing of many congenital anomaly subgroups, reducing the statistical power of the analysis [78]. Metformin may offer some benefits compared with other treatments (glyburide or insulin) during pregnancy for both mothers and their fetuses. Mothers treated with metformin for the management of GDM have been shown to have reduced gestational weight gain and less risk of gestational hypertension than those on insulin or glyburide regimens, and their offspring have a lower risk of hypoglycemia, a lower risk of macrosomia (i.e., neonatal birthweight > 4000 g), and a shorter stay in the neonatal intensive care unit (NICU), especially in the case of comparisons with glyburide [79,80]. This is probably because glyburide can directly stimulate insulin secretion from fetal pancreas, which can lead to fetal hyperinsulinism. This, in turn, can accelerate fetal growth and reduce neonatal glycemic levels immediately after delivery, increasing the likelihood of admission to NICU for the offspring born to a mother on glyburide. The effectiveness of metformin use in reducing weight gain during pregnancy has also been confirmed for obese and/or PCOS women, who are at high risk of developing GDM [81,82]. However, metformin does not preclude the rise of GDM if the drug is taken in the preconception period and during the first trimester of pregnancy in these women [83,84]. Instead, metformin seems to prevent the development of pre-eclampsia and other placental-mediated diseases (i.e., gestational hypertension) in pregnancies complicated (or not) by glucose intolerance. This clinical evidence is mostly based on a meta-analysis of 8 RCTs comparing metformin with insulin, which found a significantly lower risk of pre-eclampsia in women treated with metformin compared with those treated with insulin, with no heterogeneity among studies (RR: 0.68, 95% CI 0.48–0.95; *p* = 0.02) [85]. It is probable that this occurs because metformin reduces the expression of many inflammatory mediators, which depend upon NLRP3 inflammasome activation and contribute to the pathophysiology of placental-mediated diseases [86]. In view of these potential benefits, the extended-release formulation of metformin has been tested to prolong gestation in women with preterm pre-eclampsia in a dedicated RCT. A total of 180 women were randomized to receive 3000 mg of metformin or placebo daily, in divided doses, until delivery. The median gestational prolongation in the metformin group was 16.3 days, compared with 4.8 days in the placebo group. Based on these findings, Cluver and colleagues concluded that metformin may be used to prolong gestation in women with preterm pre-eclampsia and it can represent a novel opportunity for the successful management of this pregnancy complication [87]. Further, a meta-analysis of 35 RCTs comparing the short-term pregnancy outcomes associated with different therapies (metformin, insulin, glyburide, or placebo) found a 31% reduced risk of preterm pre-eclampsia with metformin use, but no significant difference among the treatment groups in the risk of preterm birth. However, in subgroup analyses where the indication for randomization was PCOS, metformin was associated with a lower probability of preterm birth than other treatments (*p* = 0.01). The data available were unfortunately too limited to allow for an analysis that could differentiate the risk of spontaneous versus iatrogenic preterm birth [88]. In addition, in the same meta-analysis, it was found that metformin would decrease the likelihood of caesarean section (including both elective and emergency cesarean sections) in those women who had been randomized to the treatment because of maternal obesity [88].

One potential explanation for the benefits of using metformin during pregnancy may be that the drug leads to changes in the composition of gut microbiota, which can help to reverse gut dysbiosis in GDM. Gut dysbiosis in pregnancy has been linked to various pathogenic bacteria from the Firmicutes, Proteobacteria, Bacteroidetes, and Actinobacteria phyla, including Ruminococcaceae, Desulfovibrio, Enterobacteriaceae, P. distasonis, Prevotella, and Collinsella, as well as a depletion in bacteria that produce butyrate (such as Faecalibacterium and Bifidobacterium) [89]. Although the precise mechanisms by which gut microbiota and host interact are not yet fully understood, gut dysbiosis in women with GDM has been associated with inflammation, adiposity, and glucose intolerance, and has been found to have a similar composition to that of adults with T2D [90]. Gut dysbiosis in these women tends, in fact, to persist in the postpartum period, suggesting its potential as a predictive biomarker for the development of T2D [90]. Although it has long been believed that the liver was the primary site of action for metformin, recent studies have suggested that the gut may actually be the most important site of action for this drug. This may explain why metformin so often causes gastrointestinal side effects. Metformin not only affects the gut microbiota, but also increases intestinal glucose uptake and motility, and affects bile acid turnover and intestinal release of GLP-1 [45,91,92]. Very recently, a small RCT conducted in Spain found that women with severe GDM (i.e., uncontrolled by lifestyle modifications), who were randomized to metformin therapy, had lower post-prandial glucose levels and gained less weight during pregnancy than those who were randomized to insulin therapy [93]. The metformin group also had changes in their gut microbiota, with a significant decrease in abundance of Firmicutes and Peptostreptococcaceae and a significant increase in abundance of Proteobacteria and Enterobacteriaceae. These changes in gut microbiota were inversely associated with changes in post-prandial glucose levels (for Proteobacteria) and BMI and gestational weight gain (for Enterobacteriaceae) [93]. Collectively, these data suggest that metformin may produce a beneficial clinical effect on glucose control and gestational weight gain by changing the composition of maternal gut microbiota. Several preclinical studies indicate prenatal metformin as an efficient therapy for ameliorating the transgenerational metabolic mal-programming that predisposes the offspring of mothers fed with high-fructose diets to hypertension, glucose intolerance and other metabolic morbidities [94]. Some of these transgenerational effects can be attributed to a reshaped composition of the offspring’s gut microbiota by maternal metformin [95]. With regard to humans, there is clinical evidence that breastfed infants of mothers with GDM (treated with diet plus supplemental insulin if needed) have a different microbial composition than breastfed infants of mothers without GDM [96,97]. In general, the microbial richness is lower in neonates’ stools than in the corresponding maternal samples, and fewer taxa are present in the offspring. However, infants born to GDM women show higher relative abundance of pro-inflammatory taxa (i.e., Escherichia and Parabacteroides) than infants born to healthy women, resembling the gut microbiota composition of infants born to mothers with T2D [96,97]. These results support the existence of a maternal gut microbial imprinting that is important for determining the future risk of metabolic diseases in children. It is possible that metformin, by positively affecting the gut microbiota of the mother, may exert some beneficial metabolic effects that are transmitted to the child.

Recent data suggest that in utero exposure to a diabetic, hyperglycemic environment, and the resulting experience of hypoglycemia shortly after birth, may predispose newborns of mothers with GDM to impairments in neurocognitive and psychomotor functions, possibly due to the induction of neuroinflammation. Furthermore, the chronic low-grade inflammatory state that is characteristic of maternal obesity and GDM can interfere with the correct development of the fetal adipose-brain axis, thereby resulting in long-term neurocognitive and behavioral problems, as well as predisposition to childhood obesity, in postnatal life [98]. An observational study conducted on 11–13-year-old children exposed to maternal GDM in utero found that these children had lower cortical excitability and Long-Term Depression (LTD)-like neuroplasticity than unexposed controls. This was true regardless of the pharmacological treatment (metformin and/or insulin) that the mother received for the management of GDM. The strongest predictor of these neurological phenomena, strictly linked to cortical cognitive functions, was maternal insulin resistance [99]. In another study, investigators used magnetic resonance imaging (MRI) to evaluate the potential changes related to hippocampus morphology and subfield structure, that can be caused by maternal gestational hyperglycemia, in 7–11-year-old children. It was found that the left inferior body of the hippocampus (corresponding to the CA1 subfield, a key subcortical brain area in processing emotions, memory, and learning) of GDM-exposed children had a reduced radial thickness, independently of children age, sex, and BMI z-scores [100]. These observations suggest that maternal GDM could also have negative implications for the development of limbic system functions, and thus, the emotional, memory, and learning brain networks of exposed children [98,99,100]. A follow-up study of the MIG trial found that metformin does not adversely affect neurodevelopment in children (evaluated at 2 years of age) exposed to the drug during intrauterine growth, compared with those exposed to insulin. The lowest cognitive and language scores were recorded for children born to mothers of Pacific or Indian descent, with a family history of smoking, and with unfavorable birth outcomes (i.e., macrosomia, neonatal hypoglycemia), regardless of the maternal glucose-lowering medication used for the management of GDM [101]. Similarly, any differences in weight, height, and psychomotor development in school-aged children of mothers treated with metformin versus insulin for the management of GDM, from a retrospective population-based study conducted in New Zealand, were found to be insignificant [102].

## 6. Drawbacks of Metformin Use in Pregnancies Complicated by GDM

The major limitations of using metformin to treat GDM are its potential short- and long-term negative effects on the offspring exposed to the drug during intrauterine growth. In a recent meta-analysis comparing the effects of insulin, metformin, and glyburide on neonatal biometrics [103], Tarry-Adkins and colleagues found that, although maternal glycemic control was similar for all comparisons, there was a higher rate of therapeutic failure with glyburide and metformin, requiring additional insulin treatment to maintain maternal blood glucose within the recommended targets. With regard to neonatal biometric data, these authors found that neonates exposed to glyburide had increased abdominal circumferences, while those exposed to metformin had smaller head and chest circumferences. Furthermore, neonates exposed to glyburide were heavier than those exposed to standard insulin therapy, while those exposed to metformin were lighter, with a smaller birth size and reduced lean mass [103]. Indeed, there is suggestion that metformin should not be used in cases of obstetric complications where the fetus is at risk of exposure to an ischemic environment, such as gestational hypertension or pre-eclampsia, as it may contribute to growth restriction or acidosis [76]. It is important to remember that pre-eclampsia and GDM often occur together because they share similar underlying causes, such as placental insufficiency and maternal adipose tissue dysfunction [6]. Taken all together, these data indicate that metformin, which crosses the placenta and exposes the fetus to concentrations similar to those in the maternal circulation, can negatively affect the fetal body composition and intrauterine growth. This effect is likely due to the suppression of mitochondrial respiratory chain, cellular growth, and the restriction of nutrients available for fetal metabolism, but we cannot exclude that other mechanisms may be involved [76]. This hypothesis is consistent with what has been demonstrated in preclinical studies conducted in mice, which evaluated the effect of prenatal metformin exposure on the metabolic parameters of offspring during adulthood [104]. Salomäki and colleagues found that fetuses exposed to metformin were lighter at birth, and that offsprings of both sexes were heavier from 9 weeks of age onwards, with a greater accumulation of fat at the mesenteric level when subjected to high-fat feeding. The male offspring also showed impaired glucose tolerance and elevating fasting glucose during the high-fat feeding, and down-regulated expression of GLUT4 mRNA in the epididymal fat. These findings suggest that prenatal metformin exposure can alter gene expression in the liver and impart long-term effects on the metabolic phenotype of the offspring [104]. In subsequent studies, it was reported that in the epididymal fat of male offsprings born to obese dams, in utero exposure to metformin would relate to adipocyte hypertrophy and macrophagic inflammation [105], which are known key pathogenic factors for the development of systemic insulin resistance and glucose intolerance in obese individuals [6,24]. Furthermore, while female offsprings exposed in utero to metformin delivered the same number of pups as control offsprings, the progeny litter of male offsprings was 30% smaller in size [106]. Female mice exposed to metformin in utero did not display any alterations in age of estrus onset and duration or in uterine growth and maturation, whereas the testis of male mice had smaller seminiferous tubule diameters, fewer germ cells per tubule, and fewer sperm cells in the epididymis than in control mice, with no significant motility alterations [106]. The mechanisms by which in utero exposure to metformin can have detrimental effects on male fertility and spermatogenesis are not yet known. Additionally, it is not clear if the results of preclinical studies in mice can be translated to male humans. Recently, we showed that male germ tumor cells of human origin are highly sensitive to the antiproliferative and antimigratory effects of metformin, because of the drug interferences with the expression and function of the nuclear factor HMGA1 [54], whose loss causes major histological and functional testis modifications in knock-out animals [107]. There is a general consensus that male germ cell tumors are caused by embryonic primordial germ cells migrating abnormally to the gonad during the development and are thus considered to be developmental disorders [108]. If the migrating primordial germ cells do not properly reach the gonad during this process, it could also lead to impaired male fertility, as the pool of germ cells available for adult spermatogenesis would be reduced [54,108]. As no experiments have been conducted to date in normal, non-malignant progenitor germ cells, we cannot be sure how the antiproliferative HMGA1-mediated effect of metformin could be similar to that observed in tumor cells [54]. However, these preliminary findings reinforce the need for more research to clarify the full impact of metformin on the postnatal metabolic and reproductive health of the offspring.

To date, there have been only a limited number of studies conducted on humans to determine the (potentially sex-dependent) effect of metformin on postnatal growth and metabolic phenotype of children who have been exposed in utero to the drug. The largest RCT on this topic, the MIG trial, initially found a significantly higher rate of large for gestational age (LGA) births and preterm delivery in mothers treated with metformin (plus supplemental insulin if required) than in those treated with insulin for the management of GDM [109]. However, at a mean of 7 years of age, in the follow-up cohort from Adelaide (Australia), the body composition of the children born to those two treatment groups of mothers was similar. In contrast, at a mean of 9 years of age, in the follow-up cohort from Auckland (New Zealand), the children exposed in utero to metformin were heavier and larger in several adiposity parameters, such as bodyweight, arm and waist circumference, BMI, and abdominal fat measured with MRI scan, than their insulin-exposed counterparts, independently of maternal gestational glucose control [110].

The long-term effects of metformin on children’s cardiometabolic profiles were also examined in a follow-up study of the PregMet study, an RCT comparing metformin versus placebo for the treatment of PCOS women during pregnancy [83]. The follow-up study found that children exposed to metformin have a greater BMI z-score (mean difference = 0.41, 95% CI 0.03–0.78, *p* = 0.03) which may increase the risk of cardiometabolic problems over the course of life [111]. This analysis suggests that metformin should be used with caution in pregnancy, as its long-term safety on the in utero exposed offspring is undefined. Additionally, as regards the negative maternal outcomes, metformin therapy does not prevent the development of GDM or some gestational complications, such as LGA, preterm birth, cesarean section, shoulder dystocia, perineal tear, postpartum hemorrhage, hypoglycemia, hyperbilirubinemia, or stillbirth and neonatal death in insulin resistant women with PCOS or obesity [82,83,84]. To the contrary, it may increase the risk of adverse pharmacological events, especially gastrointestinal effects (abdominal pain and diarrhea) or headache [112]. In addition, the MIG trial showed that metformin has a high failure rate for treating GDM, with 46% of women who were initially treated with metformin needing supplemental insulin to reach the glycemic target. This failure rate appears to be particularly high in cases of early diagnosis of GDM (before 25 weeks of gestation), high fasting blood glucose levels (greater than 110 mg/dl), or high A1C (greater than 5.7%). Pre-pregnancy obesity and a previous history of GDM are also predictive factors of metformin therapy failure [109]. It is worth noting that there is some debate about the minimum effective dose of metformin for treating GDM. This is not a trivial question to answer, as studies have shown that the absorption, bioavailability, and clinical benefits of the drug depend on the administered dose. Most trials on pregnant women have used a dose of metformin equal or lower than 1000 mg twice a day, mimicking the therapeutic regimens commonly used outside of pregnancy to treat T2D and other insulin resistant states. However, it has been demonstrated that women with T2D or PCOS have a lower serum concentration of metformin during pregnancy compared with the post-partum period, probably due to increased plasma volume and renal clearance, especially in mid-to late pregnancy [113,114]. Thus, it is possible that the variations in metformin pharmacokinetics, which depend on the pregnancy status of the woman, can contribute to the observed low success rates of this molecule in treating GDM (46% of women in the MIG trial received supplemental insulin therapy as add-on to metformin) [109].

One potential drawback to using metformin is the possibility of developing B12 vitamin deficiency, due to intestinal malabsorption owing to bacterial overgrowth and/or altered intrinsic factor secretion [115]. Follow-up analysis of the Diabetes Prevention Program Study has shown that long-term metformin exposure promotes B12 vitamin malabsorption and deficiency (defined by study investigators as total circulating B12 vitamin levels ≤ 203 pg/mL) in 10–30% of people. As a result, it is recommended that patients under metformin therapy have their B12 vitamin levels checked periodically and supplemented as necessary [116]. Additionally, metformin treatment may also cause a decrease in folic acid levels [117]. Both B12 vitamin and folic acid deficiencies have been linked to increased homocysteine (Hcy) concentrations [118], determining megaloblastic anemia [119], and thus can potentially exacerbate the negative consequences of a physiological gestational reduction in hemoglobin. Furthermore, these nutritional deficiencies may also give rise to neuropsychiatric disorders [116], which can heighten the emotional lability and perinatal depression that are common in pregnant women [120,121]. At present, there is inconclusive clinical evidence regarding the relationship between B12 vitamin levels, folic acid and maternal depressive symptoms or poor cognition, due to many potential endocrine interfering factors and a lack of understanding of the pathophysiological mechanisms involved (i.e., fluctuations in serotonin and dopamine neurotransmitters’ levels and/or Hcy-induced vascular injury and chronic neuroinflammation [122]). However, this is not the case for fetuses, for which there is a well-established risk of neural tube defects and other neurological disorders associated with maternal B12 vitamin and folic acid deficiencies, such as spina bifida, anencephaly and other brain malformations [118]. B12 vitamin is, indeed, essential for cognitive and neurological fetal development, impacting on memory, attention, learning, and executive functioning [123]. The central nervous system begins to develop at three weeks gestation and continues through early childhood. Myelination and synaptogenesis begin during the third trimester and continue into the first years of childhood [124]. It is important for the clinicians to be aware that if a pregnant or breastfeeding mother is deficient in vitamin B12, this can lead to deficiency in her offspring as well [118]. Such deficiency can lead to a failure in central nervous system development, even if there are no other symptoms present (such as hematological manifestations) and may remain undetected until the neurological damage is irreversible [125]. However, it should be noted that most of the data on B12 vitamin deficiency in infancy comes from case studies of infants who were exclusively breastfed by mothers who followed a vegan or vegetarian diet. The use of different diagnostic and screening criteria for GDM [4], the lack of easy-to-use, single laboratory parameters to assess B12 vitamin and/or folic acid clinical deficiency states, as well as the lack of homogeneous definitions of nutritional inadequacies [118] have led to inconsistencies in clinical study designs specifically investigating the effects of metformin on B12 vitamin and folic acid balance and child mental health. In the MIG trial, the total B12 vitamin stores (indicated by total circulating levels of B12 vitamin) were depleted during pregnancy (from randomization at 20–34 weeks of gestation to 36 weeks) to a greater extent in metformin-treated than in insulin-treated women with GDM, and the decline in B12 vitamin concentrations was more pronounced in those experiencing a longer treatment duration [126]. The concentrations of holotranscobalamin, a marker which is supposed to be indicative of the biologically active B12 vitamin’s fraction, and those of plasma Hcy, were similar between the two treatment groups of women [126].

In addition to the transgenerational potential risk of B12 vitamin and folic acid deficiencies, infants who are breastfed by mothers under metformin therapy, are also at risk of being directly exposed to the drug. Metformin is, in fact, excreted in breast milk. Although the concentration of the drug in breast milk is generally low, there is a possibility that trace amounts of the drug can pass to infants and be found in their blood circulation [127]. Therefore, it is advised to use caution when taking metformin in the early post-partum period or while breastfeeding [128].

## 7. Why and How the Guidelines on Metformin Use for GDM Differ

There is currently a great variety of recommendations in international guidelines regarding the use of metformin to treat GDM, due to ambiguous data on the short- and long-term effects of this oral drug for both mothers and offspring (Table 2). The FDA categorizes metformin as a Category B drug, meaning that studies in animals have not shown any risk to fetuses. However, there are very few RCTs on metformin use in pregnant women, and no firm conclusion can be drawn based on available data. The World Health Organization (WHO) (who.int, last accessed: 29 July 2023), the International Diabetes Federation (IDF) (idf.org, last accessed: 29 July 2023), the American College of Obstetricians and Gynecologists (ACOG) [129] and the American Diabetes Association (ADA) [130] all recommend that insulin should be the first-line pharmacological therapy in GDM management if lifestyle changes are not sufficient to reach the target glycemic levels. All these guidance authorities place metformin as a second-line pharmacological therapy in the management of GDM, proposing its use when insulin is unacceptable or unavailable for a pregnant woman. The International Federation of Gynecology and Obstetrics (FIGO) [131] recommends that insulin should be used as a first-line option in cases of diagnosing GDM before 20 weeks of gestation, when glycemic control cannot be achieved through diet and physical activity before 30 weeks of gestation, when fasting blood glucose levels are greater than 110 mg/dL, when postprandial blood glucose exceeds 140 mg/dL, or when there is a gestational weight gain over 12 kg; however, FIGO agrees on the use of metformin as a second-line pharmacological alternative to insulin in the absence of these factors [131]. The position of the Polish Diabetes Association (PDA) and Polish Society of Gynecologists and Obstetricians (PSGO) is that oral drugs should not be used for the treatment of GDM. Women taking metformin before conception are advised to switch to insulin regimens as soon as pregnancy is confirmed [132]. The guidelines set forth by the Diabetes in Pregnancy study Group India (DIPSI) recommend insulin as the first line of treatment for GDM in cases where diet alone is not sufficient (nhm.gov.in; last accessed: 29 July 2023). If the pregnant woman is unwilling or unable to take insulin, metformin may be used as an alternative starting from 13 weeks of gestation. The starting dose of metformin is 1000 mg daily, which can be escalated up to 2000 mg daily. If the maximum dose of metformin and lifestyle changes do not adequately control maternal hyperglycemia, there are no other treatment options proposed except for insulin. However, if a pregnant woman requires high doses of insulin because of severe insulin resistance, metformin may be added in order to reduce the daily insulin requirement. The Italian position is more ambiguous. In the last “Italian Standards of Medical Care 2018” document (siditalia.it; last accessed: 29 July 2023), insulin is indicated as the first-line pharmacological therapy for the treatment of GDM when, after two weeks of being diagnosed with GDM, diet and exercise are insufficient to achieve fasting and postprandial glycemic control, whereas metformin is not recommended. However, in a recent statement, the Italian Medicines Agency (AIFA) modified the summary of characteristics of extended-release metformin (Glucophage Unidie^®^) in relation to the use of this specific formulation during pregnancy and in the periconceptional period. The package insert now reads: “if clinically indicated, the use of metformin during pregnancy and in the periconceptional period can be considered in addition to or as an alternative to insulin” (agenziafarmaco.gov.it; last accessed: 29 July 2023); thereby leaving an opening for the use of metformin in GDM. After this statement, in July 2023, the Italian Diabetes Society (SID) positively evaluated the possibility of using metformin as a second-line therapy for pregnancies complicated by GDM, even though a careful and precise clinical evaluation of individual cases by an experienced diabetes specialist is advised [133]. The British National Institute for Health and Care Excellence (NICE) is the only national health authority that proposes metformin as a first-line pharmacological therapy for GDM in its guidelines (nice.org.uk; last accessed: 29 July 2023). Insulin is recommended afterwards when metformin is contraindicated or unacceptable to the woman, or when fasting plasma glucose levels are above 126 mg/dL at the time of GDM diagnosis. However, if fasting plasma glucose levels are between 108 and 125 mg/dL and there are concomitant polyhydramnios or fetal macrosomia, insulin is also recommended for use right from the start. The Society for Maternal-Fetal Medicine (SMFM) has the most divergent opinion about the safety and acceptability of insulin and metformin for pregnant women with GDM. SMFM is the first scientific society to recommend both medications as first-line pharmacological options for this condition, with metformin reported to have an efficacy and safety profile equivalent to that of insulin [134]. However, many objections were raised by GDM experts soon after publication of the SMFM statements in 2018, cautioning against the widespread adoption of metformin use during pregnancy due to its unknown effects on fetal development and programming and to the lack of controlled studies [135].

The limited availability and heterogeneity of clinical trial data account for the lack of consensus among guidelines regarding the use of metformin in pregnancy. Methodological differences (i.e., placebo-controlled vs. active comparator), varying primary outcome measurements (i.e., maternal vs. neonatal outcomes), diverse metformin dosages (i.e., ranging from 500 to 2500 mg, as per study protocols), and different types of maternal diabetes being treated contribute to this lack of agreement. Additionally, discrepancies in the clinical characteristics of the reference population of pregnant women and variation in the timing of metformin administration into gestation (starting 5 to 35 weeks of gestation) further contribute to the lack of consensus (Table 3).

The recently conducted meta-analyses by Tarry-Adkins and colleagues, as presented in Table 4, have categorized the outcomes of these studies in relation to mothers, newborns, and children [88,103]. These meta-analyses indicate that, compared with other treatments (i.e., insulin, glyburide) or placebo, metformin has either a neutral or positive effect on maternal outcomes. The results suggest that metformin is effective in reducing gestational weight gain in mothers and potentially postpartum weight retention, although it is associated with a significant risk of gastrointestinal side effects [88]. However, when metformin is used to treat GDM instead of or alongside insulin, there is an increased likelihood of low birthweight in newborns followed by accelerated growth and adipose rebound in childhood, regardless of mother’s glycemic control [103].

It is evident that additional research is required to elucidate the effects of metformin treatment on the outcomes of pregnancies complicated by GDM. Clinical trials should aim towards greater consistency in their methodologies to determine the full range of benefits and limitations associated with the use of metformin during pregnancy, as well as to identify the optimal timing and dosage for its administration in the management of GDM.

Further investigation is needed to understand the impact of metformin use in pregnant women with PCOS on childhood obesity. This exploration should also consider data from a recent population-based cohort study conducted in Sweden [142], which partially conflicts with the findings of the landmark PregMet RCT follow-up [111]. The analysis from the Swedish study revealed a positive link between maternal PCOS and childhood obesity in the offspring, with a hazard ratio of 1.61 and a 95% confidence interval of 1.44–1.81. However, no association was found between maternal metformin use and obesity in the offspring of PCOS women. Conversely, an association was observed between metformin use in non-PCOS mothers and obesity in their children. At present, there is limited clinical evidence to support specific recommendations regarding the use of metformin in pregnant women with PCOS to prevent or treat GDM [133]. Metformin shows promise in mitigating the risk of late miscarriage and preterm birth in this particular endocrine condition [84]. However, it is important to consider the potential trade-off as children born to mothers who were administered metformin during pregnancy may have higher birth weights and an increased likelihood of being overweight or obese, which indicates a potential link to unfavorable cardiometabolic outcomes in the future.

There is a necessity for a more comprehensive assessment of the impact of metformin on human embryonic and fetal tissues and its potential effects on the long-term health of the in utero-exposed offspring. This can be achieved through in vitro modeling of the hyperglycemic and pro-inflammatory conditions associated with GDM and the use of metformin in experimental culture conditions. However, obtaining authentic human embryonic and fetal tissues for such investigations is a complex issue. Additionally, immortalized human cell lines may exhibit distinct responses to metformin compared with their normal counterparts due to genetic abnormalities and altered epigenetic profiles [143]. Furthermore, the utilization and manipulation of human embryonic stem cells for differentiation into specific lineages are currently restricted by technical and ethical considerations [144].

## 8. Conclusions

Metformin is an acceptable pharmacological treatment for women with GDM not adequately controlled through medical nutrition therapy and physical activity. However, caution should be taken when using metformin during pregnancy due to its ability to pass through the placenta and the potentially detrimental effects on fetal growth and body composition. While there is some clinical data suggesting the safety of metformin for mothers and potential benefits for certain pregnancy outcomes (i.e., pre-eclampsia), further research is necessary to determine its overall impact on the long-term metabolic and reproductive health of the offspring. Prioritization of the health and well-being of both mother and child is crucial for an effective management of GDM. Consequently, if the administration of metformin increases the likelihood of obesity and T2D in the offspring, the potential advantages for the mother may be outweighed. Hence, it is appropriate to consider metformin as a suitable treatment choice for GDM solely when insulin is not a viable alternative. In the event that metformin is administered during pregnancy, it is essential to offer to women, prior to their consent, a comprehensive counseling about the contradictory evidence concerning its potential long-term safety for their offspring.

## Figures and Tables

**Table 1:** Quantitative estimates of maternal and neonatal adverse pregnancy outcomes associated with gestational diabetes mellitus (GDM) stratified by the use of pharmacological interventions for the management of the disease.

	Pharmacological Management: No (A)	Pharmacological Management: Yes (B)	Pharmacological Management: Not Reported (C)
Maternal outcomes			
Pre-eclampsia	1.39 (0.99–1.96)	1.24 (0.94–1.63)	1.46 (1.21–1.78) *
Induction of labor	1.33 (0.97–1.82)	1.83 (0.02–136.80)	1.88 (1.16–3.04) *
Instrumental delivery	0.83 (0.02–41.94) *	0.52 (0.20–1.33)	1.10 (0.59–2.06)
Caesarean section	1.16 (1.03–1.32)	1.70 (0.99–2.90)	1.38 (1.20–1.58) *
Shoulder dystocia	1.26 (0.98–1.62)	1.29 (0.87–1.92)	1.48 (0.99–2.20)
Premature rupture of membranes	–	–	1.13 (1.06–1.20) *
Postpartum hemorrhage	1.07 (0.68–1.66)	–	0.94 (0.75–1.17)
Neonatal outcomes			
Stillbirth	1.08 (0.38–3.11)	–	0.78 (0.58–1.05)
Congenital malformation	0.78 (0.36–1.70)	1.62 (0.65–4.07)	1.18 (1.10–1.26) *
Pre-term birth	1.51 (1.26–1.80) *	1.22 (0.99–1.50)	1.51 (1.19–1.93) *
Respiratory distress syndrome	1.38 (0.76–2.50)	1.57 (1.19–2.08) *	1.59 (0.89–2.83)
Low 1′ APGAR score	1.43 (1.01–2.03)	1.63 (0.88–3.02)	–
Low 5′ APGAR score	1.11 (0.74–1.66)	0.94 (0.70–1.27)	1.12 (0.96–1.32)
Macrosomia	1.70 (1.23–2.36) *	1.56 (0.92–2.66)	1.48 (1.13–1.95)
LGA birth	1.57 (1.25–1.97) *	1.61 (1.09–2.37) *	1.42 (0.98–2.06)
Low birthweight	1.40 (1.12–16.73)	0.87 (0.53–1.43)	0.94 (0.84–1.05)
SGA birth	0.83 (0.55–1.23)	0.77 (0.46–1.29)	1.19 (0.67–2.11)
Neonatal hypoglycemia	3.94 (0.37–42.05)	–	11.71 (7.49–18.30) *
Neonatal jaundice	1.33 (0.94–1.88)	1.28 (1.02–1.62) *	–
Admission to NICU	1.34 (0.86–2.09)	2.29 (1.59–3.31) *	2.28 (1.26–4.13) *

Data are presented as odds ratio (OR) estimates, adjusted for potential confounding factors (i.e., maternal age, pre-pregnancy body mass index, gestational weight gain, gravidity, parity, smoking history, and chronic hypertension). Values in brackets indicate the 95% Confidence Interval for each OR. Pharmacological interventions refer to the use of insulin only. Column A includes studies in which GDM women had never used insulin during the course of the disease; column B includes studies in which different proportions of GDM women were treated with insulin; column B includes studies in which insulin use was not reported [2]. Asterisks (*) denote statistical significance. LGA: large for gestational age; SGA: small for gestational age; NICU: neonatal intensive care unit.

**Table 2:** Guidelines for the pharmacological management of GDM that is not adequately controlled with medical nutrition therapy and physical activity.

Society	First Line	Second Line
ACOG	Insulin	Metformin
ADA	Insulin	Metformin or glyburide
IDF	Insulin	Metformin
WHO	Insulin	Metformin
FIGO	Insulin	Metformin
PDA/PSGO	Insulin	None
DIPSI	Insulin	Metformin
SID	Insulin	None *
NICE	Metformin	Insulin
SMFM	Insulin or metformin	Glyburide

ACOG: American College of Obstetricians and Gynecologists; ADA: American Diabetes Association; WHO: The World Health Organization; IDF: International Diabetes Federation; FIGO: International Federation of Gynecology and Obstetrics; PDA: Polish Diabetes Association; PSGO: Polish Society of Gynecologists and Obstetricians; DIPSI: Diabetes in Pregnancy study Group India; SID: Italian Diabetes Society; NICE: The British National Institute for Health and Care Excellence; SMFM: Society for Maternal-Fetal Medicine. * Starting from July 2023, SID has opened to the potential administration of extended release metformin in pregnancy, within specific and individualized situations [133].

**Table 3:** Summary of key clinical trials investigating the effects of metformin administration during pregnancy on mothers and/or their children.

Study	Sample Size	Study Population Characteristics	Metformin Daily Dosage	Timing of Metformin Administration	Primary Study Outcome(s)
MIG [109], RCT	N = 751 women (N = 363, metformin group; N = 388, insulin group)	Singleton pregnant women diagnosed with GDM according to ADIPS criteria	500 to 2500 mg (plus supplemental insulin, if needed)	Starting 20 to 33 weeks of gestation	Perinatal outcome: a composite of neonatal hypoglycemia, respiratory distress, need for phototherapy, birth trauma, 5-min Apgar score <7, or prematurity (RR 1.00, 95% CI 0.90–1.10, ns)
MIG TOFU [136], Post-RCT follow-up	N = 318 children (N = 154, children born to metformin-treated women; N = 164, children born to insulin-treated women)	Children (aged 2 years), born to mothers randomized in the MiG-trial			Children outcome: body composition measured with anthropometry, bioimpedance, and DXA (larger mid-upper arm circumferences, and larger subscapular and biceps skinfolds in the metformin-exposed group)
MIG TOFU [110], Post-RCT follow-up	N = 208 children (N = 103, children born to metformin-treated women; N = 105, children born to insulin-treated women)	Children (aged 7–9 years), born to mothers randomized in the MiG trial			Children outcome: body composition measured with anthropometry, bioimpedance, DXA, and MRI (larger measures of weight, arm and waist circumferences, waist-to-height ratio, BMI, triceps skinfold, fat mass and lean mass, abdominal fat volume in the metformin-exposed group from the Auckland cohort, no differences between groups in the Adelaide cohort)
ISRCTN10845466 [137], RCT	N = 106 women (N = 53, metformin group; N = 53, placebo group)	Singleton pregnant women diagnosed with GDM according to Malaysian national criteria	1000 to 1500 mg (plus supplemental insulin, if needed)	Starting 16 to 30 weeks of gestation	Maternal outcome: change in A1c at 36 weeks of gestation (mean A1c increment +0.20% vs. +0.27%, ns)
MiTy [138], RCT	N = 502 women (N = 233, metformin group; N = 240, placebo group)	Singleton pregnant women with pregestational T2D	2000 mg (as add-on to insulin)	Starting 6 to 22 weeks of gestation	Perinatal outcome: a composite of pregnancy loss, preterm birth, birth injury, moderate/severe respiratory distress, neonatal hypoglycemia, or neonatal intensive care unit admission longer than 24 h (RR 1.02, 95% CI 0.83–1.26, ns)
PregMet [83], RCT	N = 257 women (N= 136 metformin group, N = 240 placebo group)	Singleton pregnant women with PCOS	2000 mg	Starting 5 to 12 weeks of gestation	Maternal outcomes: prevalence of pre-eclampsia (risk difference 3.7%, 95% CI −1.7–9.2, ns), GDM (risk difference 0.8%, 95% CI −8.6–10.2, ns), preterm delivery (risk difference −4.4%, 95% CI, −10.1–1.2, ns), and a composite of these three endpoints (risk difference 1.5%, 95% CI −8.9–11.3, ns)
PregMet 2 [84], RCT	N = 487 women (N= 244 metformin group, N = 243 placebo group)	Singleton pregnant women with PCOS	2000 mg	Starting 6 to 12 weeks of gestation	Maternal outcomes: frequency of late miscarriage and preterm birth (5% vs. 10%, odds ratio 0.5, 95% CI 0.22–1.08, ns), incidence of GDM (25% vs. 24%, odds ratio 1.09, 95% CI 0.69–1.66, ns)
PedMet [111], Post-RCT follow-up	N = 141 children (N = 71, children born to metformin-treated women; N = 70, children born to placebo-treated women)	Children (aged 5–10 years), born to mothers randomized in the PregMet trial			Children outcomes: BMI Z score (difference in means +0.41, 95% CI 0.03–0.78)
NCT01240785 [139], RCT	N = 221 women (N= 111, metformin group; N = 110, insulin group)	Singleton pregnant women with GDM diagnosed according to Finnish national criteria	1000 to 2000 mg (plus supplemental insulin, if needed)	Starting 22 to 34 weeks of gestation	Perinatal outcomes: birthweight expressed in grams (difference in means +15, 90% CI −121–89, ns), birthweight > 90 centile (RR 0.9, 95% CI 0.5–1.8, ns)
UMIN 000005393 [140], RCT	N = 94 women (N = 47, metformin group; N = 47, insulin group)	Singleton pregnant women with GDM diagnosed according to Carpenter-Coustan criteria	1700 to 2550 mg	Starting 28 to 34 weeks of gestation	Maternal outcomes: gestational weight gain (mean Kg, 0.53 vs. 2.3), frequency of pre-eclampsia (21.7% vs. 15.2%, ns), preterm birth (10.9% vs. 10.9%, ns), caesarean section (71.7% vs. 65.2%, ns)
IRCT201306057841N4 [141], RCT	N = 119 women (N = 59, metformin group; N = 60, insulin group)	Singleton pregnant women with GDM diagnosed according to ADIPS criteria	500 to 1500 mg (plus supplemental insulin, if needed)	Starting 25 to 35 weeks of gestation	Perinatal outcomes: caesarean section (74% vs. 70%, ns), neonatal hypoglycemia (ns), Apgar score (ns), birthweight (mean grams, 3176 vs. 3342, ns)
EMPOWaR [82], RCT	N = 449 (N = 226 metformin group, N = 223 placebo group)	Singleton pregnant women with obesity according to WHO criteria and without GDM	500 to 2500 mg	Starting 12 to 16 weeks of gestation	Perinatal outcome: Z score of birthweight percentile (difference in means −0.029, 95% CI −0·217–0.158, ns)

**Table 4:** Summary of meta-analyses investigating the comparative effects of metformin administration during pregnancy in relation to alternative treatment options.

Meta-Analysis Outcomes	Summary of Results
Maternal outcomes ^§^	Lower gestational weight gain (mean difference 1.57 kg ± 0.60 kg) Lower risk of any hypertensive disease of pregnancy (including pre-eclampsia) (odd ratio 0.76, 95% CI 0.60–0.95) Higher risk of gastrointestinal side-effects (odds ratio 2.43, 95% CI 1.53–3.84) No difference in risk of preterm birth (spontaneous or iatrogenic) (odds ratio 0.90, 95% CI OR 0.67–1.21) No difference in risk of delivery by caesarean section (odds ratio 0.90, 95% CI 0.82–1.00) Lower risk of maternal hypoglycemia (odds ratio 0.47, 95% CI 0.28–0.80)
Neonatal outcomes ^‡^	Lower neonatal birthweight (mean difference −73.92 g, 95% CI −114.79–−33.06 g) Lower risk of neonatal macrosomia (OR 0.60, 95% CI 0.45–0.79) No difference in neonatal abdominal circumference (mean difference 0.00 cm, 95% CI −0.44–0.44 cm)
Children outcomes ^‡^	At 2 years of age Higher infant bodyweight (mean difference 440 g, 95% CI 50–830) No difference in infant height (mean difference 0.65 cm, 95% CI −1.31–2.61) At 5–9 years of age No difference in childhood bodyweight (mean difference 1.13 kg, 95% CI −0.19–2.45) No difference in childhood height (mean difference 0.02 cm, 95% CI −1.46–1.50) Higher childhood BMI (mean difference 0.78 kg/m^2^, 95% CI 0.23–1.33)

^§^ Metformin vs. other treatments or placebo for any indication of administration (i.e., PCOS, obesity, or management of GDM) [88]; ^‡^ Metformin vs. insulin for the management of GDM [103].

## Data Availability

Not applicable.
